# Modeling the impact of high temperatures on microalgal viability and photosynthetic activity

**DOI:** 10.1186/s13068-017-0823-z

**Published:** 2017-05-26

**Authors:** Quentin Béchet, Martin Laviale, Nicolas Arsapin, Hubert Bonnefond, Olivier Bernard

**Affiliations:** 1 Université Côte d’Azur, Inria, BIOCORE, BP 93, 06902 Sophia Antipolis Cedex, France; 2 Sorbonne Universités, UPMC Université Paris 06, CNRS, UMR 7093, LOV, Observatoire océanologique, 06230 Villefranche/Mer, France

**Keywords:** *Dunaliella salina*, Biofuel, Viability, Heat stress, Thermal dose, Productivity, Outdoor cultivation

## Abstract

**Background:**

Culture collapse due to high temperatures can significantly impact the profitability of outdoor algal cultivation systems. The objective of this study was to model for the first time the impact of high temperatures on algal activity and viability.

**Results:**

Viability measurements on *Dunaliella salina* cultures were based on cytometry with two fluorescent markers (erythrosine and fluorescein di-acetate), and photosynthetic activity was measured by Pulse Amplitude Modulation (PAM) fluorometry. Kinetic studies revealed that viability and activity losses during exposure to high temperatures could be described by a Weibull model. Both mortality and activity were shown to be functions of the thermal dose received by the algae, defined as the product of duration of exposure to high temperatures and an exponential function of temperature. Simulations at five climatic locations revealed that culture collapse due to high temperatures could impact productivity of *D. salina* in non-temperature-controlled outdoor photobioreactors by 35 and 40% in arid and Mediterranean climates, respectively.

**Conclusions:**

The model developed in this study can be used to forecast the impact of high temperatures on algal biofuel productivity. When coupled with models predicting the temperature of outdoor cultivation systems, this model can also be used to select the best combination of location, system geometry, and algal species to minimize the risks of culture collapse and therefore maximize biofuel productivity.

**Electronic supplementary material:**

The online version of this article (doi:10.1186/s13068-017-0823-z) contains supplementary material, which is available to authorized users.

## Background

Massive investments were done on microalgae industry in the last decades, mainly due to their capacity to synthesize lipids for biofuel production [1]. The economic feasibility of this new biotechnology at full-scale has been the object of a large number of studies [2–4] but remains difficult to accurately evaluate, mainly because of uncertainties on the actual algal productivity that can be reached at full-scale (i.e., biomass produced per unit time per square meter of installation).

Mathematical models have been developed to predict and optimize the biofuel production potential of microalgae as a function of local climate (light intensity, temperature, etc.) and process operation (retention time, nutrients concentration, etc.) [5–7]. Regarding the impact of temperature, existing models are able to accurately estimate productivity when temperature is within a range of values enabling algal growth [8]. However, temperatures of typical cultivation systems (i.e., photobioreactors, open ponds) can exceed these temperatures. For example, Torzillo et al. [9] observed that the temperature in a photobioreactor located in Florence, Italy, reached levels higher than 40 °C for several hours per day in summer. Tredici and Materassi [10] even observed temperatures as high as 56 °C in vertical alveolar panels that caused the collapse of the thermotolerant *Spirulina* sp. In these conditions, heat stress impacts structure and activity of proteins and membrane fluidity, which disturbs metabolic processes and leads to retardation in growth [11, 12]. Cooling the system is then necessary to avoid culture collapses but strongly increases operation costs at full-scale [13]. As culture collapses would have a dramatic impact on the system profitability and environmental footprint, models predicting the impact of heat stress on algal productivity are needed to accurately assess full-scale biofuel production [14].

Several studies aimed to understand the impact of heat stress on microalgae [15], and especially on microalgae symbiotic with coral [16] and microphytobenthos [17–20]. For example, Vieira et al. [20] showed that the photosynthetic activity of two microphytobenthos communities significantly decreased during continuous exposure to a temperature of 42 °C. The work of Serra-Maia et al. [21] also highlighted the impact of temperature on cell mortality in photobioreactors exposed to high temperatures for several days. However, to the best of our knowledge, no previous study systematically measured the evolution of photosynthetic activity (i.e., the rate of electron transfer in algal photosystems) during heat stress over short time-scales (from minutes to hours) for various temperatures. In addition, the rate at which algae die when exposed to high temperatures was, to the best of our knowledge, never measured. From what temperature do algae start to die? How long can algae survive when exposed to lethal temperatures? To answer these questions, the objective of this study was to develop a model for predicting algal photosynthetic activity and viability (i.e., the fraction of living cells in the culture) when algae are exposed to high temperatures. For this purpose, the effect of short-term (<3 h) heat exposure (between 41 and 60 °C) on the photosynthetic activity and viability of the commercial species *Dunaliella salina* were studied. *D. salina* is a species which has been studied for its potential to produce both carotenoids and triacylglycerols (TAG) which can be turned into biofuel [22]. To quantify the impact of high temperature on productivity at full-scale, the resulting viability and activity models were coupled with a model predicting temperature fluctuations in outdoor photobioreactors at various climatic locations.

### Review on existing mortality models

To the best of our knowledge, the short-term impact of high temperatures on the viability and photosynthetic activity of microalgae has not been previously modeled. However, multiple models exist to predict the mortality rate of bacteria under various stresses: high/low pH stress [23], high temperatures [24], high pressure [25], etc. This section reviews the mortality models developed for bacteria with the objective to select the most relevant model to describe the impact of high temperatures on microalgae.

Several formulas were used in the literature to describe the survival rate of bacteria exposed to heat stress (see the reviews [26–28]). One of the most traditional approaches is based on a first-order model, assuming a constant mortality rate *m* (s^−1^), expressed as follows:

1 $$N(t) = N_{0} \exp ( - mt),$$

where *N* is the number of viable cells at the time *t* (s) and *N*_0_ the number of viable cells at *t* = 0 s. This model has been largely criticized in the literature due to its inability to represent experimental data, and especially the initial lag-phase usually observed at the start of mortality events [28–31], which was also observed in this study (see “Results” section). The model presented by Geerard et al. [27] aimed to better represent this initial lag-phase and was expressed as a set of two differential equations:

2 $$\frac{{{\text{d}}N}}{{{\text{d}}t}} = - m_{\text{m}} N\left( {\frac{1}{{1 + C_{\text{c}} }}} \right)\left( {1 - \frac{{N_{\text{res}} }}{N}} \right)$$

3 $$\frac{{{\text{d}}C_{c} }}{{{\text{d}}t}} = - m_{\text{m}} C_{\text{c}},$$

where *C*_c_ is a variable representing the “physiological state” of cells, *m*_m_ is the maximal decay rate (s^−1^), and *N*_res_ is a model parameter. A simpler model, the ‘log-logistic model’ proposed by Cole et al. [32], was used in numerous studies to represent the impact of various stresses on bacterial viability [25, 30, 33–35]:

4 $$N(t) = N_{0} \exp \left( {\alpha + \frac{\omega - \alpha }{{1 + \exp \left( {\frac{4\sigma (\tau - \log (t))}{\omega - \alpha }} \right)}}} \right),$$

where *α* and *ω* are, respectively, the viable cell counts at the start and at the end of the mortality event (in log values), *σ* is a shape factor, and *τ* is a scale factor. This model was in agreement with experimental data but was specifically designed to represent the case where the final cell count is different from 0 (for example, in the case of bacterial resistance to stress). As described in the “Results” section, no algae survived after heat treatment in our kinetic studies and this model was therefore not adapted. Finally, the other commonly used model in the literature is the Weibull model, described as

5 $$N(t) = N_{0} \,\exp \left( { - \left( {\frac{t}{\lambda }} \right)^{n} } \right),$$

where *λ* is the half-life parameter (s) and *n* is the shape factor (*λ* is the time of exposure necessary to kill 63% of the population; low *n* values indicate a sharp decrease of the viability over time). Van Boekel [29] reported 55 studies that successfully used the Weibull model to represent viability loss during heat treatment of various bacteria. This model is able to represent the initial lag-phase at the start of heat treatment, has a limited number of parameters, and is practical to use. Based on this literature review, the Weibull model was selected to represent the decrease of activity and viability of algae during exposure to high temperatures.

## Methods

### Algal species and cultivation conditions

The commercial species *D. salina* (CCAP 18/19) was cultivated in f/2-enriched seawater medium [36]. An axenic culture (volume 300 mL) was maintained at 27 ± 1 °C under continuous light (300 μmol m^−2^ s^−1^). Homogenous mixing of the culture was ensured by bubbling filtered air (PTFE filters 0.2 µm, Midisart 2000, Sartorius) combined with gentle magnetic stirring. Bubbling also removed excessive oxygen and supplied inorganic carbon. The culture was operated in a semi-continuous mode by replacing twice a day a fraction of the culture by freshly prepared medium, thereby maintaining the algal concentration at approximately 7 × 10^5^ cells mL^−1^.

### Kinetic studies of algal activity and viability at high temperatures

The algal cell density was determined using a particle counter (HIAC-Royco; Pacific Scientific Instruments). Variability between triplicate measurements was routinely less than 5%. The algal culture was then diluted with 0.2 µm-filtered f/2 medium to reach a concentration of around 10^4^ cells mL^−1^, which was found optimal for both activity and viability measurements. Several 1 mL aliquots of this diluted culture was placed in 1.5 mL centrifuge tubes (Safe-Lock tubes; Eppendorf AG, Germany) and immersed in a water bath preheated at the desired temperature (41, 42, 43, 45, 50 or 60 °C). The thermal inertia of the samples was very low due to their reduced volume, and thus the desired temperature was very rapidly reached. The time necessary for cooling was however in the same order of magnitude than the time of exposure to 60 °C (maximum of 3 min), which may have impacted the accuracy of measurements as discussed in the “Results” section. While this sudden temperature drop may have impacted cell activity and viability, this was necessary to control the exposure time to temperature during experiments. To avoid potential issues resulting from the reduced sample volume, algae were highly diluted and kept in the dark when exposed to heat, which avoided inhibition or limitation by oxygen, inorganic carbon, or nutrients during the incubation time. At least six different exposure times were tested for each temperature (from 30 s to 3 h depending on the tested temperature) to estimate the evolution of viability and activity during the course of high-temperature exposure. Inhibition by excessive oxygen concentration or growth limitation by limiting carbon supply was therefore unlikely during heat stress. Sedimentation was not observed in viable samples, mostly because *D. salina* cells are motile. It is therefore unlikely that experimental conditions significantly impacted viability or activity of algal cells. At the end of each exposure time, tubes were immediately placed in a colder water bath (20 °C), still in dark conditions. Viability measurements through cytometry were performed 1 and 6 h after the end of heat exposure as described below (for example, for the kinetics study at 41 °C, tubes were first exposed to a temperature of 41 °C for up to 3 h, and then viability was measured 1 and 6 h after the end of heating). As for the estimation of photosynthetic activity, the tubes were first cooled down to 20 °C before being transferred to a 27 °C water bath (i.e., the temperature used for cultivating the algae) and in dim light until PAM analysis was performed within 1 h after the heat exposure.

### Assessment of microalgal viability by flow cytometry

Various experimental techniques have been proposed in the literature and a definition of a “viable cell” depends on the technique used. For example, the “viable cell count” measures the fraction of cells able to produce a single colony on an Agar plate, while some staining techniques rely on the ability of dead/living cells to absorb a certain dye [37]. In this study, two fluorescent markers were used to measure viability by cytometry (BD Accuri™ C6 Plus): erythrosine (Erythrosin extra-bluish, CAS: 16423-68-0, Sigma-Aldrich, USA), which stains algal cells with a porous cell membrane (i.e., dead cells [38]), and fluorescein di-acetate (FDA, CAS: 596-09-8, Sigma-Aldrich, USA), which stains algal cells having enzymatic activity (i.e., living cells [39]).

The fluorescent markers were added to the algal samples by adding a small volume of concentrated markers (erythrosine: 20 μL mL^−1^_culture_ at 1 g L^−1^_sea water_ filtered at 0.2 µm; FDA: 3 μL mL^−1^_culture_ at 10 mg mL^−1^_acetone_; marker concentrations were optimized to ensure that viable and non-viable cells could be clearly identified; data not shown). For measurements with erythrosine, samples were exposed for 60 min to the marker in the dark. For measurements with FDA, samples were kept for 20 min at room temperature (20 °C) and in the ambient light (measured within 20–50 μmol m^−2^ s^−1^) to stimulate enzymatic cell activity of living cells. Cells fluorescence was measured 1 and 6 h after heat exposure by cytometry on three different fluorometry channels. Chlorophyll a fluorescence was measured by using the “FL3” channel (Excitation/Emission: 488/>670 nm) and enabled distinguishing algae from bacteria and/or other non-photosynthetic particles. “FL2” (Excitation/Emission: 488/585 nm) and “FL1” (Excitation/Emission: 488/530 nm) were used to detect the fluorescence of erythrosine and FDA, respectively. The fractions of living and dead cells were determined from erythrosine/FDA fluorescence vs. Chlorophyll fluorescence plots (see Additional file 1: S1 for an illustration).

**Fig. 1:**
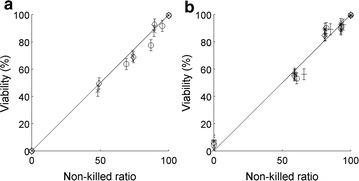
Viability of *D. salina* cultures for different killed and non-killed ratios for erythrosine (**a**) and FDA (**b**) and for different incubation times (Erythrosine: *crosses* 15 min; *diamonds* 2 h; *circles* 3 h; FDA: *crosses* 6 min; *diamonds* 21 min; *circles* 36 min; *stars* 51 min; ‘*plus*’ *signs* 67 min). *Error bars* represent 95% confidence intervals

This viability measurement protocol was validated on samples with known ratios of viable and non-viable cells. For this purpose, a sample from the culture of *D. salina* was heated at 45 °C for 1 h in order to kill all microalgae (i.e., 0% viability). Known volumes of this sample were mixed with another non-killed sample (i.e., close to 100% viability) in order to obtain the following theoretical fractions of killed algal cells: 0, 25, 50, 75, and 100%. The viability of each of these samples was then measured for different incubation times with erythrosine and FDA (Fig. 1). In practice, the total cell concentration in the heated solution was lower than in the non-heated solution due to cell degradation during heating (microscopic observations; data not shown). The ratios of killed and non-killed cells in each sample were therefore re-calculated by determining from cell counts the number of cells degraded during heating. The significant linear correlations shown in Fig. 1 (*R* ^2^ = 0.99, *N* = 15 for erythrosine and *R* ^2^ = 0.98, *N* = 25 for FDA) for the two viability markers indicate that the technique developed in this study enabled accurate measurements of *D. salina* viability.

### Assessment of microalgal photosynthetic activity by in vivo chlorophyll fluorescence analysis

Chlorophyll fluorescence was measured by Pulse Amplitude Modulation (PAM) fluorometry, which has been shown to be a useful technique for assessing the effect of temperature on photosynthetic activity [12, 15, 20]. The fluorescence signal was measured with a Multi-Color PAM (Heinz Walz GmbH, Germany) equipped with a temperature-controlled block for cuvette set at 27 °C ± 1 °C, a RG665 long-pass filter on the fluorescence detector, a blue LED (440 nm) as source of actinic light, and a white LED used as a light source for saturating pulses [40]. For each tube exposed to heat, a 500 µL-aliquot was transferred into a quartz cuvette (QS-10, Hellma Analytics) before being diluted with fresh medium (total volume of the cuvette: 1.25 mL) to avoid cell mutual shading during PAM measurement. A so-called “rapid light curve” (RLC) protocol was then applied [41]. For this purpose, each sample was exposed for 5 min to a light intensity of 22 µmol m^−2^ s^−1^, which corresponds to the first light step of the RLC, to ensure that all samples could experience the same short-term light history, i.e., that photosystems were activated and that the chlorophyll fluorescence signal reached steady-state [42]. The sample was then exposed to 7 successive 10 s steps of increasing actinic light: 22, 79, 218, 467, 812, 1336, and 1890 µmol m^−2^ s^−1^. A saturating light pulse was applied at the end of each step and the instantaneous and maximum light-acclimated fluorescence levels (*F* and *F*_M_ *’*, respectively) were measured. Thus the effective quantum yield of photosystem II (*ΦPSII*) was calculated for each light step according to Genty et al. [43]:

6 $$\varPhi {\rm{PSII}} = \frac{{F_{\text{M}}^{\prime} - F}}{{F_{\text{M}}^{\prime }}}$$

For each sample, a *Φ*PSII-*I* curve (i.e., rapid light curve [41]) was thus obtained, where *I* is the instantaneous photosynthetically active radiation (PAR: 400–700 nm, in µmol m^−2^ s^−1^), which was previously measured inside the cuvette with a spherical micro quantum sensor (US-SQS/L, Walz). According to Silsbe and Kromkamp [44], each *ΦPS*II-I curve was then fitted to the model of Eilers and Peeters [45]. The ‘Eilers and Peeters’ model, when reparametrized as suggested by [8], can be characterized by the following parameters: the photosynthetic efficiency at low light intensity (i.e., *α*, the initial slope of the curve), the maximal rate of photosynthesis (i.e., the maximal value of the curve plateau), and the light intensity threshold between photosaturation and photoinhibition [46]. All photosynthetic parameters varied significantly with heat exposure (see Additional file 1: S2 for details). Among them, the initial slope of the curve *α* was chosen as the best indicator of the algal photosynthetic activity as it was estimated with the highest level of confidence (see Additional file 1: S2 for an illustration).

### Model calibration and statistical analysis

Each kinetic study (i.e., the evolution of photosynthetic activity and viability during heating) was used to fit the Weibull model by least-square regression (Matlab function *lsqcurvefit*). Viability and photosynthetic activity were measured in duplicates for each time of exposure. Confidence intervals on measured algal viabilities and activities were based on a statistical analysis of the differences between duplicate values. The uncertainty on model parameters was then estimated through Monte Carlo simulations based on these confidence intervals, as detailed in Additional file 1: S3.

### Prediction of the impact of high temperature on full-scale cultivation

Simulations were performed to determine the impact of high temperatures in outdoor photobioreactors at various climatic locations, based on the model of viability and activity developed in this study. Simulations were performed at five climatic locations representing arid, Mediterranean, subtropical, tropical, and temperate climates as described in [13]. The temperature prediction was coupled to the models of algal activity and viability described in the “Results” section. Unless otherwise stated, it was assumed that the photobioreactor was re-inoculated with fully viable algae at sunrise the day following a culture collapse. The number of culture collapses to high temperature was defined as the number of days when viability at the end of the day was lower than 1%.

## Results

### Impact of high temperatures on *D. salina* viability

**Fig. 2:**
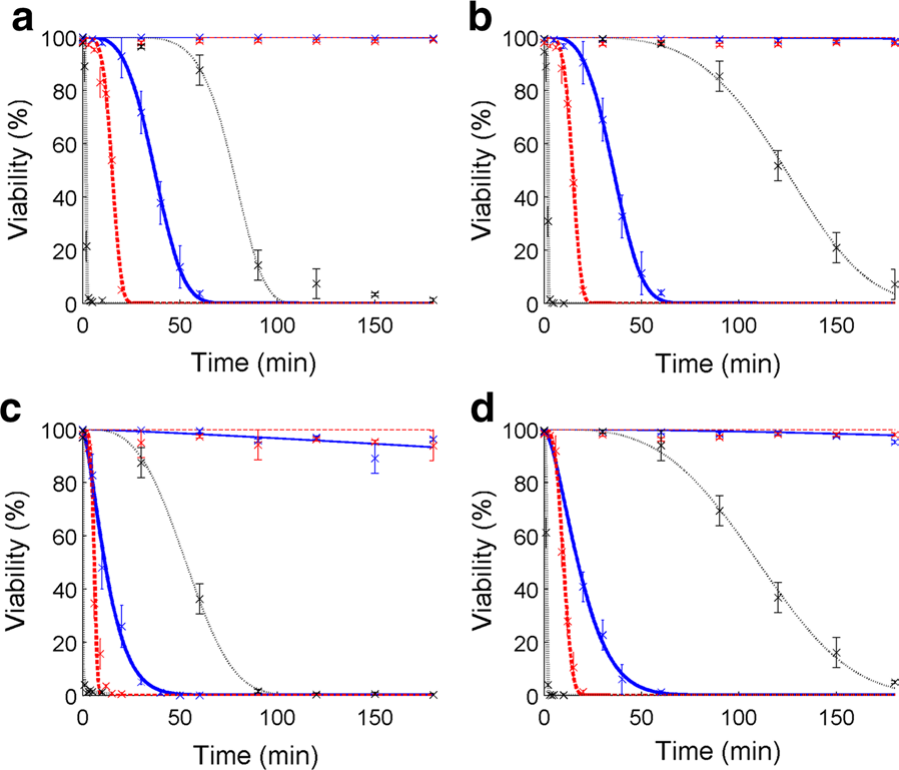
Evolution of *D. salina* viability with time of exposure to high temperatures (Marker/Incubation time: **a** erythrosine/1 h; **b** FDA/1 h; **c** erythrosine/6 h; **d** FDA/6 h; *crosses* experimental data; *line* Weibull model; *Thin blue line T* = 41 °C; *Thin red dash line T* = 42 °C; *Thin black point line T* = 43 °C; *Thick blue line T* = 45 °C; *Thick red dash line T* = 50 °C; *Thick black point line T* = 60 °C). *Error bars* represent 95% confidence intervals. The 100% viability measured in the tubes non-exposed to heat (*t* = 0) shows that experimental conditions (no agitation, dark conditions) did not impact algal viability over the duration of kinetic studies

Figure 2 shows that the viability of *D. salina* did not significantly decrease for temperatures lower than 43 °C (values below 100% viability in Fig. 2 for 41 and 42 °C are most likely due to experimental uncertainty). This confirms that cell viability was not impacted by the test experimental conditions (i.e., tubes were kept in the dark and without agitation). The protocol used in this study therefore enables studying the impact of temperature stress only while allowing for low thermal inertia, on the contrary to previous protocols described in the literature (e.g., Serra-Maia et al. [21]). Above 43 °C, the rate of viability loss increased with temperature, which is consistent with previous results reported in literature for bacteria [29]. This increase can be explained by the fact that algal death is most likely due to degradation of key enzymes and membrane denaturation, the rate of which follows an Arrhenius function of temperature [29]. Interestingly, viability measured with erythrosine (Fig. 2a–c) decreased slightly faster with the time of heat exposure than the viability measured with the FDA marker (Fig. 2b–d). Erythrosine is adsorbed by dead cells due to membrane permeability [47], whereas FDA is absorbed by cells and then hydrolyzed by enzymes of viable cells, thus resulting in the production of a fluorescent compound. The differences in the rates of viability loss between erythrosine and FDA therefore indicate that algal cell membrane became permeable slightly before enzymatic activity stopped. In addition, Fig. 2 shows that viability measured 6 h after exposure to high temperatures was significantly lower than viability 1 h after heat exposure. This decrease is unlikely due to slow penetration of erythrosine in dead cells as Béchet et al. [47] showed that only a few minutes were necessary for erythrosine to enter dead algal cells. This decrease therefore suggests that algae continued dying after exposure to high temperatures. Algal death due to high temperatures can therefore be considered as a two-step process: a fast decrease of viability during heat exposure followed by a slower decrease after heat exposure.

**Fig. 3:**
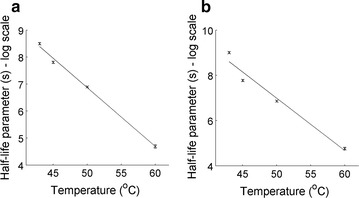
Evolution of Weibull *λ* parameter (Eq. 5) with temperature when erythrosine (**a**) and FDA (**b**) were used to measure *D. salina* viability 1 h after heat exposure (see Table 1 for parameters values). Results obtained for measurements performed 6 h after heat exposure are shown in Additional file 1: S5. *Error bars* represent 95% confidence intervals

The measured rates of viability loss during heat exposure are consistent with previous observations from the literature: firstly, Fig. 2 shows that the Weibull model was able to represent the evolution of algal viability with the time of heat exposure, which was observed for diverse microorganisms (see the “Background” section for details; see Additional file 1: S4 for models comparison). Secondly, Fig. 3 shows that the half-life parameter *λ* (Eq. 5) followed an exponential function of temperature, which was reported by most of the 55 studies reviewed by van Boekel [29]:

7 $$\lambda = \exp (a_{\text{V}} (T - T_{{0,{\text{V}}}} )),$$

where *a*_v_ is the exponential coefficient (°C^−1^). Thirdly, this coefficient *a*_V_ (Eq. 7) determined for *D. salina* was between −0.218 and −0.222 °C^−1^ (Table 1), which is within the range reported by van Boekel [29] for diverse microorganisms (−0.05 to −0.37). Finally, the shape factor ‘*n*’ did not follow a clear evolution with temperature (data not shown), which suggests that variations of *n* are likely due to experimental errors. This observation is also consistent with the findings of van Boekel [29] who showed that the shape parameter was independent of temperature for the large majority of the microorganisms tested. Based on these similarities, mechanisms responsible for algal and bacterial deaths due to exposure to high temperatures are likely to be similar. Following these observations, the viability model was expressed as follows:

**Table 1:** Parameters values for the Weibull model (values in parenthesis are 95% confidence intervals; model parameters shown in this table were obtained with erythrosine when viability was measured 1 h after heat exposure; see Additional file 1: S5 for parameters obtained 6 h after heat exposure)—see the “Results” section for parameters definition

	Marker	*a*_V_ */a*_A_ (°C^−1^)	*T*_0,V_ */T*_0,A_ (°C)	Shape factor *n*
Viability	Erythrosine	−0.218 (±0.004)	81.5 (±0.7)	4.84 (±1.18)
	FDA	−0.232 (±0.004)	80.1 (±0.6)	5.59 (±3.10)
Activity	–	−0.132 (±0.001)	92.2 (±0.4)	3.97(±0.20)

8 $$V(t) = V_{0} \exp \left( { - \left( {\frac{t}{{\exp (a_{\text{V}} (T - T_{{0,{\text{V}}}} )}}} \right)^{{n_{\text{V}} }} } \right),$$

where *V* is the viability; *V*_0_ the initial viability; *T*_*0,*V_ (°C) and *a*_V_ (°C^−1^) are obtained from log-linear regressions as shown in Fig. 3; and *n*_V_ is obtained as the average of experimental measurements of the shape parameter at different temperatures (Table 1).

Interestingly, Eq. 8 suggests that viability is a function of the ‘dose’ of heat, or ‘thermal dose’ (*d*_V_, s), which can be defined as:

9 $$d_{V} = t \exp ( - a_{\text{V}} (T - T_{{0,{\text{V}}}} )),$$

where *t* (s) is the duration of exposure to the temperature *T* (°C). The concept of ‘thermal dose’ has already been used in the medical literature to represent the impact of high temperatures on the viability of tissue cells and was expressed using the same mathematical equation [48]. This type of expression suggests that viability of algal samples exposed to changing temperatures can be determined by integrating the dose *d*_V_ over the period of time considered:

10 $$V(t) = V_{0} \exp ( - (d_{\text{V}} )^{{n_{\text{V}} }} ).$$

**Fig. 4:**
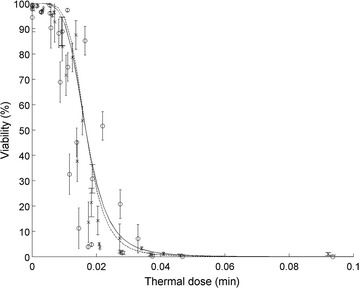
Evolution of the viability with the thermal dose as defined by Eq. 9 (*Crosses*/*plain line* measurements/prediction with erythrosine; *Circles*/*dash-line* measurements/prediction with FDA)—results obtained for measurements performed 6 h after heat exposure are shown in Additional file 1: S5. *Error bars* represent 95% confidence intervals

Figure 4 shows that the Weibull model associated with the concept of thermal dose (Eq. 10) successfully predicted the evolution of algal viability with time for all temperatures tested.

### Impact of high temperatures on *D. salina* photosynthetic activity

**Fig. 5:**
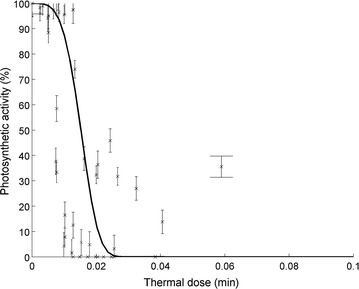
Evolution of the measured (*crosses*) and predicted (*plain line*) photosynthetic activity (characterized here by the slope of rapid light curves at low light intensities) with the thermal dose. Experimental data include the triplicates at 45 °C. *Error bars* represent 95% confidence intervals

Similarly to what was observed for *D. salina* viability, the Weibull model was able to represent the evolution of algal photosynthetic activity with duration of heat exposure (see Additional file 1: S6). Photosynthetic activity decreased faster than viability, which indicates that photosynthesis logically stopped before algal death. Consequently, accounting for the impact of high temperatures on viability only may lead to overestimate productivity as cells stop photosynthesis before dying. Moreover, the half-life parameter *λ* for photosynthetic activity (Eq. 5) was shown to increase exponentially with temperature (Additional file 1: S6) and the shape factor *n* was not clearly correlated to temperature (data not shown). Following the same approach than for algal viability, the following equations were used to represent the activity drop during heat exposure:

11 $${A}(t) = {A}_{0} \exp ( - (d_{A} )^{{n_{A} }} )$$

12 $$d_{A} = \exp (a_{A} (T - T_{{0,{A}}} ),$$

where* A* is the photosynthetic activity;* A*_0_ the initial photosynthetic activity; *d*_*A*_ the thermal dose for photosynthetic activity (s); and *a*_*A*_ (s^−1^) and *T*_0,*A*_ and *n*_*A*_ were defined similarly for the viability model (Eq. 8). The poor fit shown in Fig. 5 can be explained by the uncertainty on the temperature in test tubes (due to tubes thermal inertia) during exposure to high temperatures at these short time-scales (minutes).

### Accounting for viability loss in algal growth models

Equations 9, 10, 11, and 12 can be used to express algal viability and photosynthetic activity when algae are exposed to constant temperatures. The objective of this section is to propose a modeling strategy to express viability and photosynthetic activity when algae are exposed to fluctuating temperatures, for example, in outdoor cultivation systems. The modeling approach proposed here consists on the following steps:

1. The temperature profile experienced by the algal culture should be first determined over the entire cultivation period to identify mortality events (i.e., the time periods at which temperature is above the lethal value; e.g., 43 °C for *D. salina*).
2. For each mortality event, the fractions of viable and active cells (*f*_V_ and *f*_A_, respectively), at the end of the event should be determined by using the following equations:

13 $$f_{\text{V}} = \exp ( - d_{\text{V}}^{{n_{\text{V}} }} ),$$

14 $$f_{\text{A}} = \exp ( - d_{\text{A}}^{{n_{\text{A}} }} ),$$

where *n*_V_ and *n*_A_ are given in Table 1 and *d*_V_ and *d*_A_ are the thermal doses defined as follows:

15 $$d_{\text{V}} = \mathop \int \limits_{{t_{\text{s}} }}^{{t_{\text{e}} }} { \exp }( - a_{\text{V}} (T(t^{\prime}) - T_{{0,{\text{V}}}} ) \cdot {\text{d}}t^{\prime},$$

16 $$d_{\text{A}} = \mathop \int \limits_{{t_{\text{s}} }}^{{t_{\text{e}} }} \exp ( - a_{\text{A}} (T(t^{\prime}) - T_{{0,{\text{A}}}} ) \cdot {\text{d}}t^{\prime},$$

where *t*_s_ and *t*_e_ are the starting and end times of the mortality event, respectively (s); *T* is the time-dependent culture temperature (°C); and *T*_*0,*V_ and *T*_*0,*A_ (°C) are the parameters given in Table 1.

3. The active and viable algal biomass at the end of the mortality event can then be computed by multiplying the active and viable biomass concentrations before the mortality event by the two fractions *f*_A_ and$f_{{{\text{V}}}}$.
4. When temperature is below the lethal level (43 °C for *D. salina*), the evolution of biomass concentration in the system can be predicted by using classical models, generally expressed by the following differential equation [5–7]:

17 $$\frac{{{\text{d}}N}}{{{\text{d}}t}} = \phi (T)\mu (f_{\text{i}} )N,$$

where *N* is the algal cell concentration (m^−3^); *Φ* is a function representing the impact of temperature on the algal growth rate; *μ* is the specific growth rate (s^−1^); and *f*_i_ represents the factors other than temperature impacting algal growth rate (light intensity, pH, nutrient concentrations, etc.).

An important assumption behind this modeling approach is that non-active algae (i.e., algae which activity dropped to 0 after heat exposure) cannot recover after heat exposure. This assumption may, however, not be valid for some algal species such as some benthic algae which were observed to recover after an exposure to 50 °C [19]. Accounting for this recovery process could therefore refine the modeling approach, even if out of the scope of this study. The impact of this assumption on productivity predictions in outdoor cultivation systems is discussed in the following section.

## Discussion

### Impact at full-scale

To demonstrate the impact of high temperatures on full-scale algal cultivation systems for biofuel production, the following simulations were performed. The temperature profile in tubular vertical outdoor photobioreactors (radius 0.095 m; height 1.8 m) was predicted by the validated model of Béchet et al. [49]. This model is based on a heat balance considering various heat fluxes reaching outdoor photobioreactors: solar heat flux, long-wave radiative fluxes, convection, etc. Model parameters and various assumptions were described by Béchet et al. [49]. The viability and photosynthetic activity models were then coupled with these predictions to determine the fractions of viable and active cells over 1 year of operation. The potential coupled impact of high light and high temperature was not accounted for in these simulations, simply because to the best of our knowledge, there is no model available to predict this coupled impact on algal viability. The simulations discussed in this section only aim to provide an estimation of the impact of high temperature only on biofuel production in outdoor cultivation systems.

**Fig. 6:**
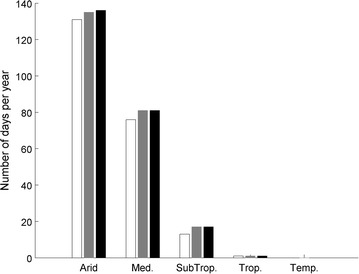
Number of days when viability (*white bars*) and photosynthetic activity (*gray bars*) was lower than 1% at the end of the day, and when temperature reached values higher than 43 °C at least once during the day (*dark bars*)—model parameters for the viability model obtained with erythrosine and 1 h after exposure to high temperature (see Table 1 for details)

Figure 6 shows that mortality events leading to culture collapse would happen 76 and 131 days per year in Mediterranean and arid climates, respectively. These simulations were based on the model parameters obtained with erythrosine 1 h after exposure to high temperature (see Table 1 for details). Very similar results were obtained when using the model parameters obtained with other sets of parameters (obtained with FDA and/or 6 h after heat exposure; data not shown). This indicates that the variation of model parameters caused by the different experimental techniques during model calibration only caused a small level of uncertainty on the viability predictions in outdoor photobioreactors. Moreover, the number of days when photosynthesis was completely de-activated did not differ by more than 4 days from the number of days when algae died (Fig. 6). This low difference is due to thermal inertia of the closed photobioreactors used in this study. When temperature reached a level of 43 °C, the culture temperature indeed stayed above this lethal temperature for at least 1 h, leading to full loss of both viability and photosynthetic activity. This result indicates that the assumption that de-activated algae cannot recover after thermal stress does not significantly impact productivity predictions in photobioreactors as photosynthesis de-activation is almost automatically followed by algal death.

Because of the practical necessity to grow an inoculum to re-inoculate outdoor photobioreactors, it was assumed that photobioreactors can be re-inoculated 5 days after a culture collapse. Under this assumption, algae could not be cultivated during a significant number of days in photobioreactors located in a Mediterranean or arid climate. The model predicted that between 35 and 40% of the light reaching the photobioreactors would be absorbed by dead algal cells in these two climates. Based on the assumption that biofuel productivity is proportional to the amount of light captured, mortality events can negatively impact the yearly biofuel productivity of outdoor photobioreactors by approximately 35 and 40% in Mediterranean and arid climates, respectively.

### Choice of the species

Based on the frequent occurrence of culture collapses predicted by the viability model, cultivating *D. salina* is not recommended in Mediterranean and arid climates without temperature control. These conclusions, however, only apply to the case of column photobioreactors having the same geometry than the reactors considered in this study. For example, increasing the reactor radius, and thus its thermal inertia, could lead to minimize temperature fluctuations and therefore avoid regular mortality events. The model developed in this study, when coupled to temperature-predicting models, can therefore be used as an optimization tool for system design to maximize biofuel production. In addition, while this study focused on the algal species *D. salina*, the same approach could be applied to other algal species, such as *Spirulina platensis,* which is known to resist to high temperatures [9, 10], or other species more adapted for biofuel production. Unfortunately, many potential biofuel producers, such as *Nannochloropsis* sp. and *Phaeodactylum tricornutum*, are marine species which result from billion years of selection in an environment where temperature is generally below 30 °C. The optimal growth temperature of these species is therefore usually below 30 °C [8], and mortality rates may already be important at these temperatures. This model can therefore be adapted and used to determine the best algal species and/or select the optimal location to maximize algal biofuel productivity.

### Other model applications

Modeling the impact of high temperatures on microalgae could have many applications in the study of natural ecosystems such as coral–microalgae symbiosis [16] and estuarine microphytobenthos communities [17]. For example, Laviale et al. [18] measured temperatures as high as 42 °C during summer in top sediment layers in intertidal flats in Portugal (Southern Europe Atlantic coast). Photosynthesis de-activation and even mortality events are therefore likely to happen in microphytobenthos communities. Predicting the impact of these high temperatures on algal photosynthetic activity and viability may be the key to further understand the coupled effects of light and heat stress on these microorganisms. In addition, because of global warming and the subsequent temperature rise in oceans, important rates of algal mortality may occur in marine environments. Considering the high importance of phytoplankton in the food chain, the modeling approach developed in this study may help assessing the impact of global warming on marine ecosystems.

## Conclusions

- Both algal viability and photosynthetic activity of *D. salina* were significantly affected above a temperature threshold of 43 °C, and their responses over time of exposure to heat were shown to follow a 2-parameters Weibull-like model.
- Algal viability and photosynthetic activity were both shown to be functions of the thermal dose, defined as the product of time and an exponential function of temperature.
- The application of the viability and activity models coupled with a physical model predicting temperature fluctuations in closed photobioreactors revealed that cultivating *D. salina* for biofuel production in this type of cultivation systems is non-viable in arid and Mediterranean climates due to the high occurrence of culture collapses.
- In the first approximation, the number of culture collapses can be assumed to be equal to the number of days when temperature exceeds the maximal temperature for algal activity and viability.
- When coupled with models predicting temperature in outdoor cultivation systems, the biological model developed in this study can be used to optimize the combination of algal species, location, and system geometry to maximize system profitability.
- Out of the context of biofuel production, the model developed in this study could have many other applications in natural ecosystems.

## Additional file

**Additional file 1: S1.** Cytometry analysis. **S2.** An example of Pulse Amplitude Modulation (PAM) fluorometry analysis. **S3.** Uncertainty analysis via Monte-Carlo simulations. **S4.** Comparison of Weibull and first-order fits to experimental data. **S5.** Viability results 6 hours after heat exposure. **S6.** Evolution of the photosynthetic activity during kinetic studies.
