# Supplementary material for: Modeling the impact of high temperatures on microalgal viability and photosynthetic activity
Source: Biotechnol Biofuels. 2017 May 26;10:136. doi: 10.1186/s13068-017-0823-z (PMC5446765; doi:10.1186/s13068-017-0823-z)
Supplement: Supplementary file 1 — Additional file 1: S1. Cytometry analysis. S2. An example of Pulse Amplitude Modulation (PAM) fluorometry analysis. S3. Uncertainty analysis via Monte-Carlo simulations. S4. Comparison of Weibull and first-order fits to experimental data. S5. Viability results 6 hours after heat exposure. S6. Evolution of the photosynthetic activity during kinetic studies. [file 13068_2017_823_MOESM1_ESM.docx]

Modeling the impact of high temperatures on microalgal viability and photosynthetic activity - Supporting information

Quentin Béchet^a,b^, Martin Laviale^a,b^, Nicolas Arsapin^a,b^, Hubert Bonnefond^a,b^, Olivier Bernard^a,b,*^

^a^ Université Côte d’Azur, Inria, BIOCORE, BP 93 06902 Sophia Antipolis Cedex, France

^b^ Sorbonne Universités, UPMC Université Paris 06, CNRS, UMR 7093, LOV, Observatoire océanologique, F-06230, Villefranche/mer, France

# Cytometry analysis

This supporting information aims to illustrate how cytometry results were analyzed to determine cell viability.

It was first necessary to distinguish algal cells from other particles such as cell debris, bacteria or inorganic solids. Algal cells are generally bigger than other particles in samples and the chlorophyll content of particles is higher in algae than in other particles. Algal populations were therefore identified from 'Size vs. Chlorophyll content' plots (see Figure S1-S1 for an illustration)


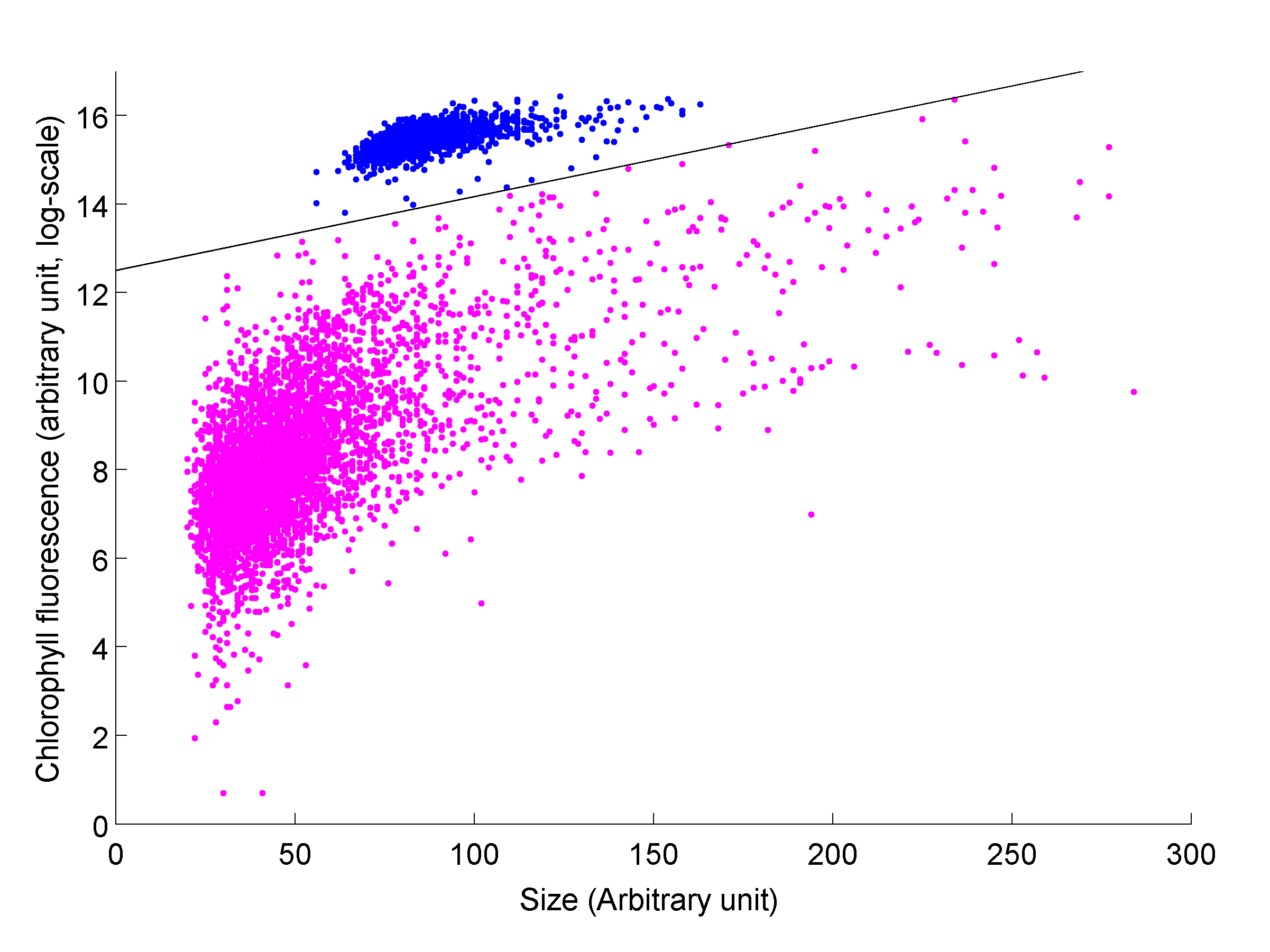


Figure S1-S1: Point-plot showing the size and Chlorophyll fluorescence of each particle analyzed by the cytometer (each point corresponds to a single particle; blue dots: algal cells; Purple dots: other particles; black-line: separation between algal cells and other particles) - Sample of *Dunaliella salina* exposed to a temperature of 50^o^C for 9 min, analysis performed 1 hour after heat exposure.

Distinction between viable and non-viable cells was performed by measuring the fluorescence of algal cells at different wavelengths corresponding to the emission wavelength of two dyes: erythrosine and fluorescein di-acetate (FDA). As explained in the main manuscript, viable cells were stained by FDA and non-viable cells were stained by erythrosine. In practice, viability was measured by direct observations of 'Chlorophyll content vs. dye fluorescence' as illustrated in Figures S1-S2 and S1-S3.


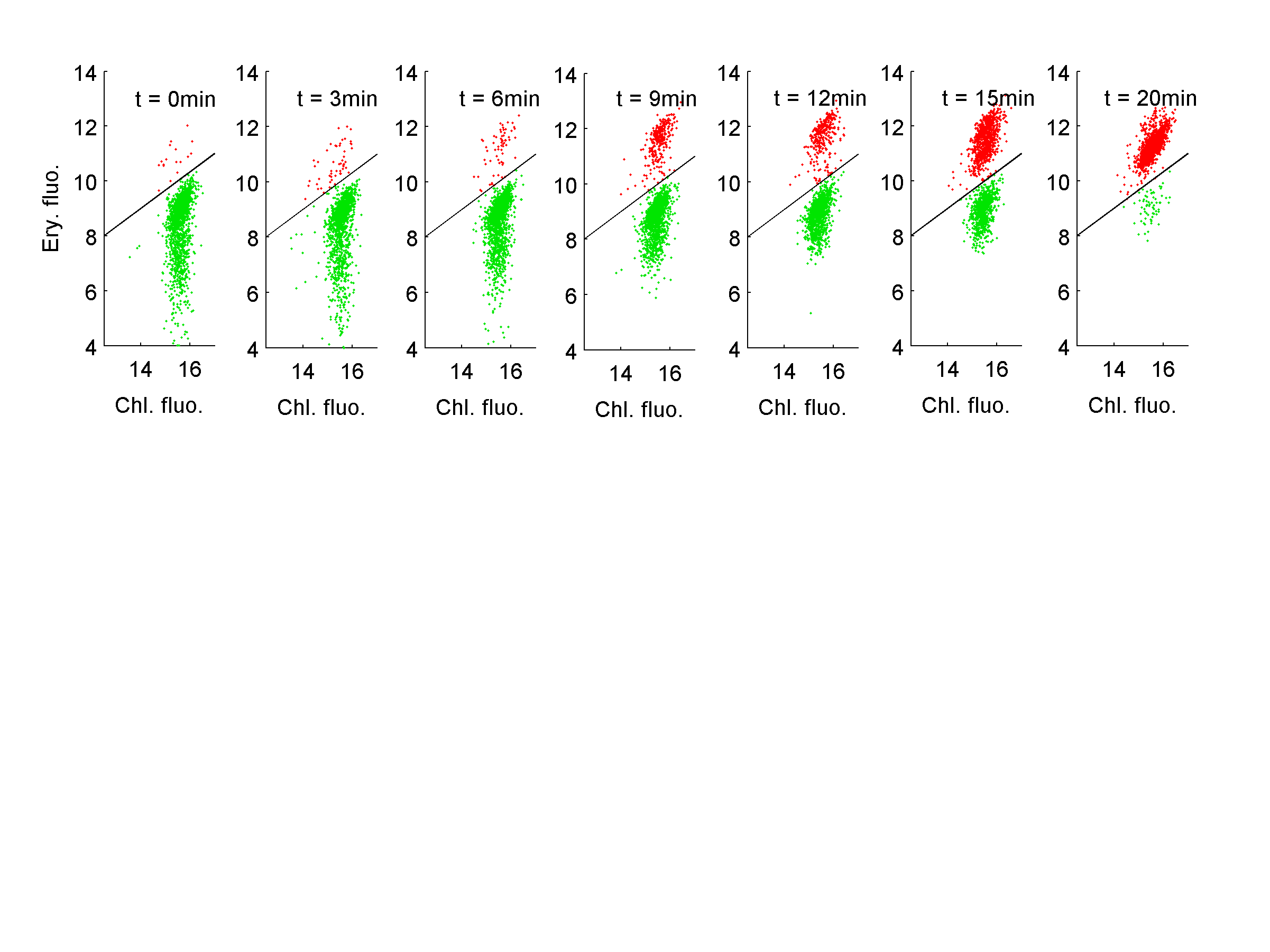


Figure S1-S2: Point-plot showing the fluorescence of erythrosine and Chlorophyll content of each particle analyzed by the cytometer (each point corresponds to a single particle: green points: viable algal cells; red points: non-viable algal cells; black line: separation between viable and non-viable cells) - Sample of *Dunaliella salina* exposed to a temperature of 50^o^C; times of exposure are shown on each sub-plot, analysis performed 1 hour after heat exposure.


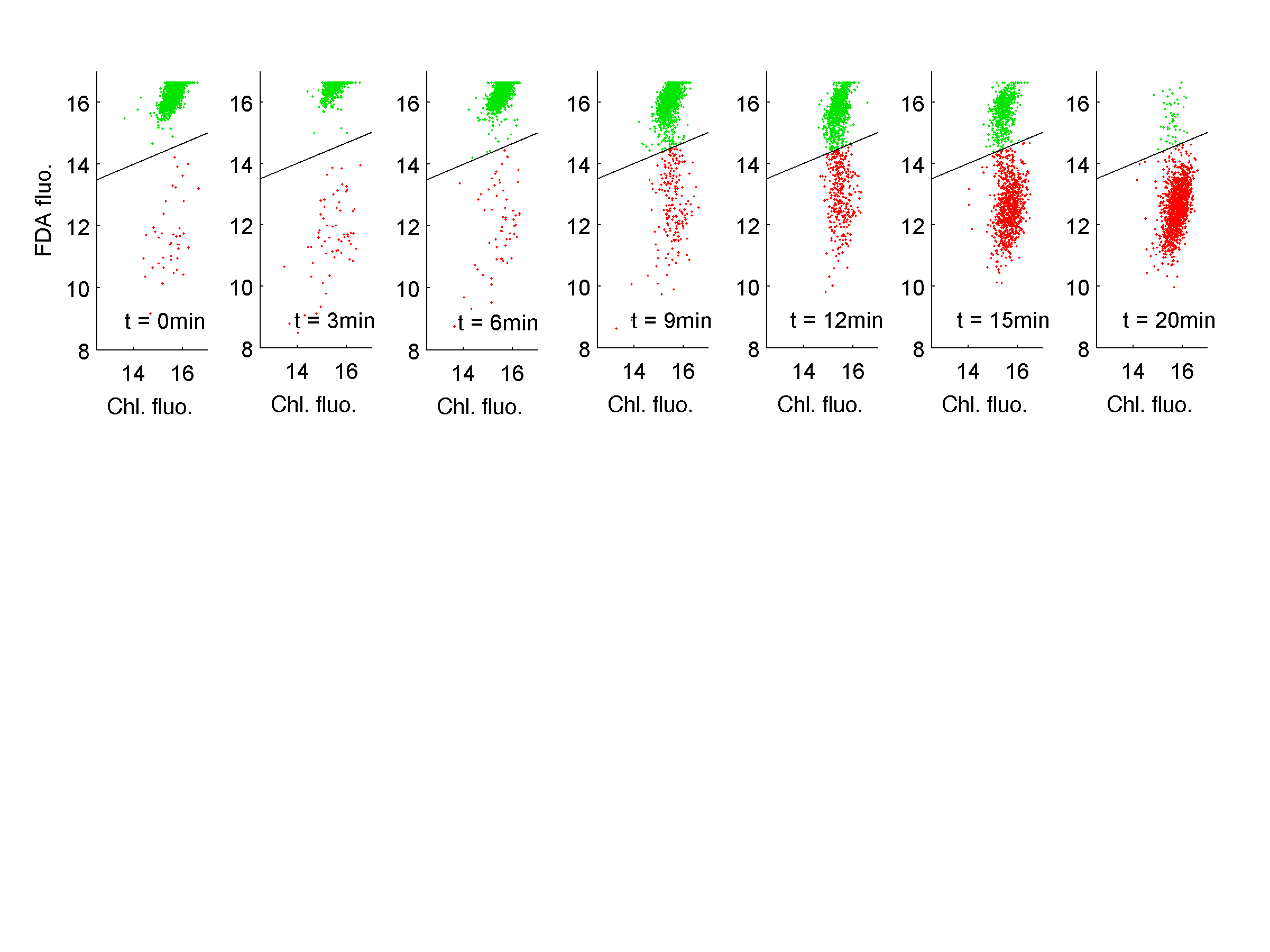


Figure S1-S3: Point-plot showing the fluorescence of FDA and Chlorophyll content of each particle analyzed by the cytometer (each point corresponds to a single particle: green points: viable algal cells; red points: non-viable algal cells; black line: separation between viable and non-viable cells) - Sample of *Dunaliella salina* exposed to a temperature of 50^o^C; times of exposure are shown on each sub-plot, analysis performed 1 hour after heat exposure.

# An example of Pulse Amplitude Modulation (PAM) fluorometry analysis

This supporting information aims to illustrate how the "rapid light curves" obtained by pulse amplitude modulation (PAM) were used to determine the photosynthetic activity of algal samples.

Figure S2-S1 shows the rapid light curves obtained during heat exposure of a *D. salina* sample at 50^o^C. After a heat exposure of several minutes, the model of Eilers and Peeters was unable to fit the experimental light response, simply because algal activity was fully inhibited by heat. In practice, the photosynthetic efficiency was identified from fitting the Eileers and Peeters model using a least-square fitting regression (*lsqcurvefitting* Matlab function) at the start of kinetic studies and was then taken as 0 when the model was unable to fit the data. The resulting values of the photosynthetic efficiency at low light intensity (α, the initial slope of the *ΦPSII* –I curve) and the maximal photosynthetic activities are shown in Figure S2-S2 and S2-S3, respectively.


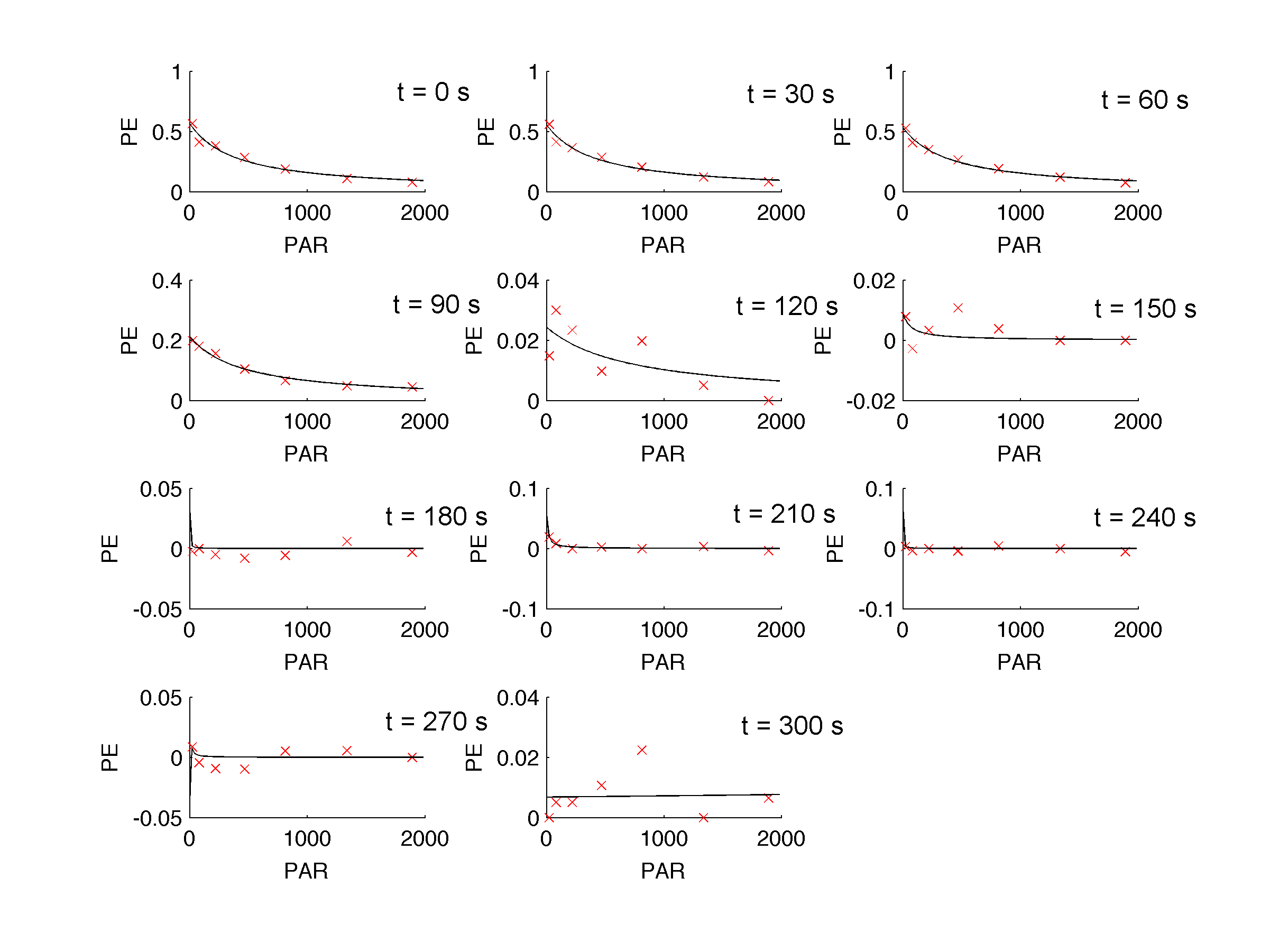


Figure S2-S1: Evolution of the rapid light curve with time of exposure (up to 300 s) of *D. salina* at 50°C (PE: Photosynthetic efficiency, symbolized by *ΦPSII* in the main manuscript; PAR: Photosynthetically active radiation in μmol m^-2^ s^-1^). Red crosses: experimental data (ΦPSII). Solid lines: Eilers and Peeters model fit.


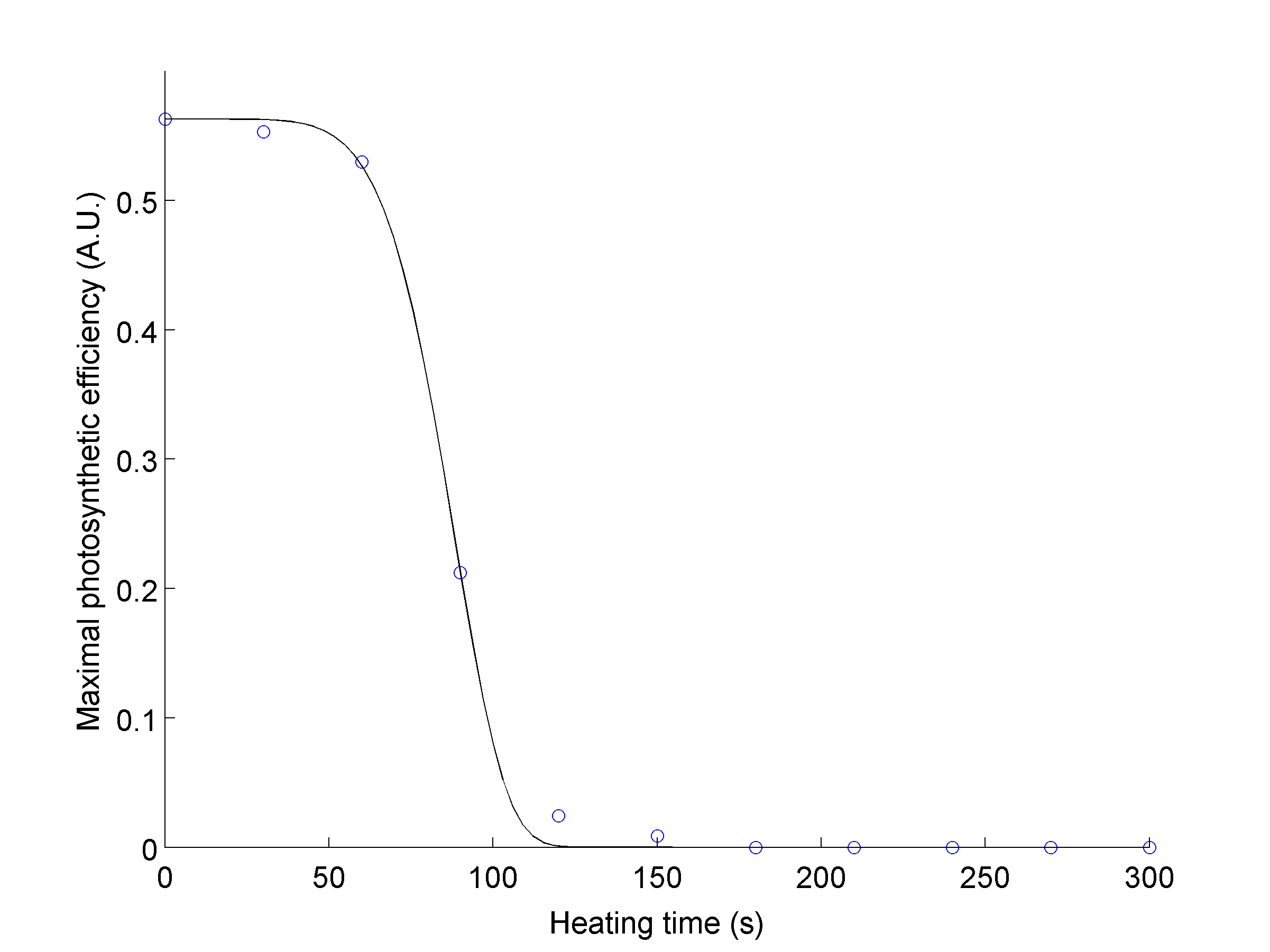


Figure S2-S2: Evolution of the photosynthetic efficiency at low light intensity (α, the initial slope of the *ΦPSII* –I curve) over time of *D. salina* at 50^o^C (Circles: experimental data; Line: Weibull fitting)


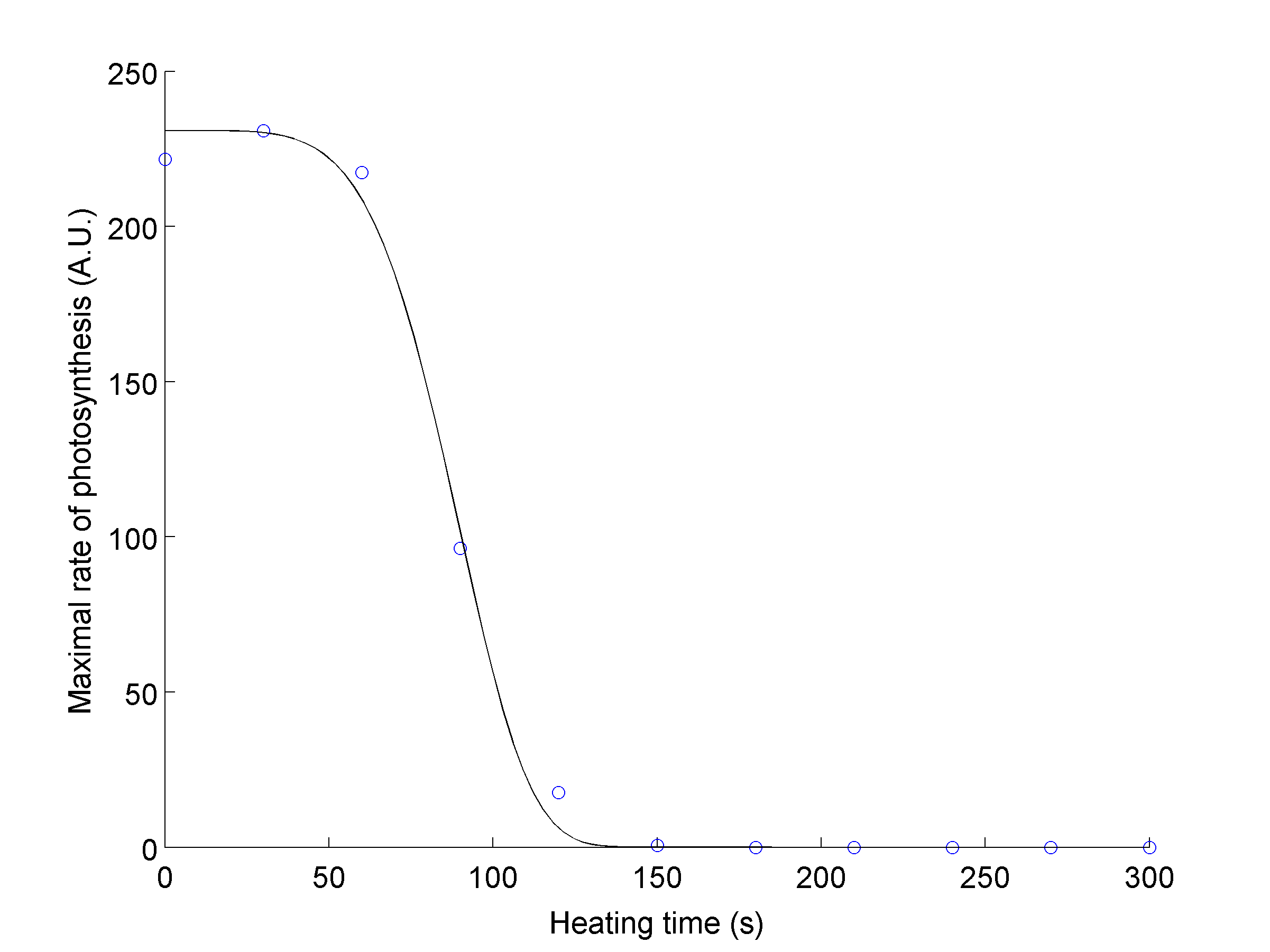


Figure S2-S3: Evolution of the maximal rate of photosynthesis over time of *D. salina* at 50^o^C (Circles: experimental data; Line: Weibull fitting).

# Uncertainty analysis via Monte-Carlo simulations

The inaccuracies on viability and photosynthetic activity measurements during kinetic studies caused uncertainty on model parameters. This supporting information aims to detail how Monte-Carlo simulations were used to determine confidence intervals on model parameters.

## Viability

The viability of 226 algal samples was measured in duplicates during the kinetic studies presented in the main manuscript. Figure S3-S1 shows that the error (defined here as the difference between two replicates) tends to be relatively small when viability was close to 0% or 100%, but higher for mid-range values. These variations are explained by the fact that it was more difficult to distinguish stained vs. non-stained algal cells on cytograms when algae are only partially dead (see S1 for details). For this reason, the samples were separated in two distinct groups: samples for which viability was close to 0% or 100% (i.e. between 0 and 5% and 95% and 100%) and samples for which the viability was close to mid-range values (i.e. between 5% and 95%). The error distribution for these two groups was found to be normal as shown in Figures S3-S2 and S3-S3. Consequently, the absolute value of error followed a half normal distribution, which mean *μ_HN_* can be expressed as a function of the standard deviation of the normal distribution as follows:

$$\mu_{HN}=\frac{\sigma\sqrt{2}}{\sqrt{\pi}}$$

The standard deviations of the error for these two groups were then estimated as follows:

- *σ* = 0.65 % when viability was either between 0-5% or between 95-100%
- *σ* = 5.76 % when viability was between 5-95%


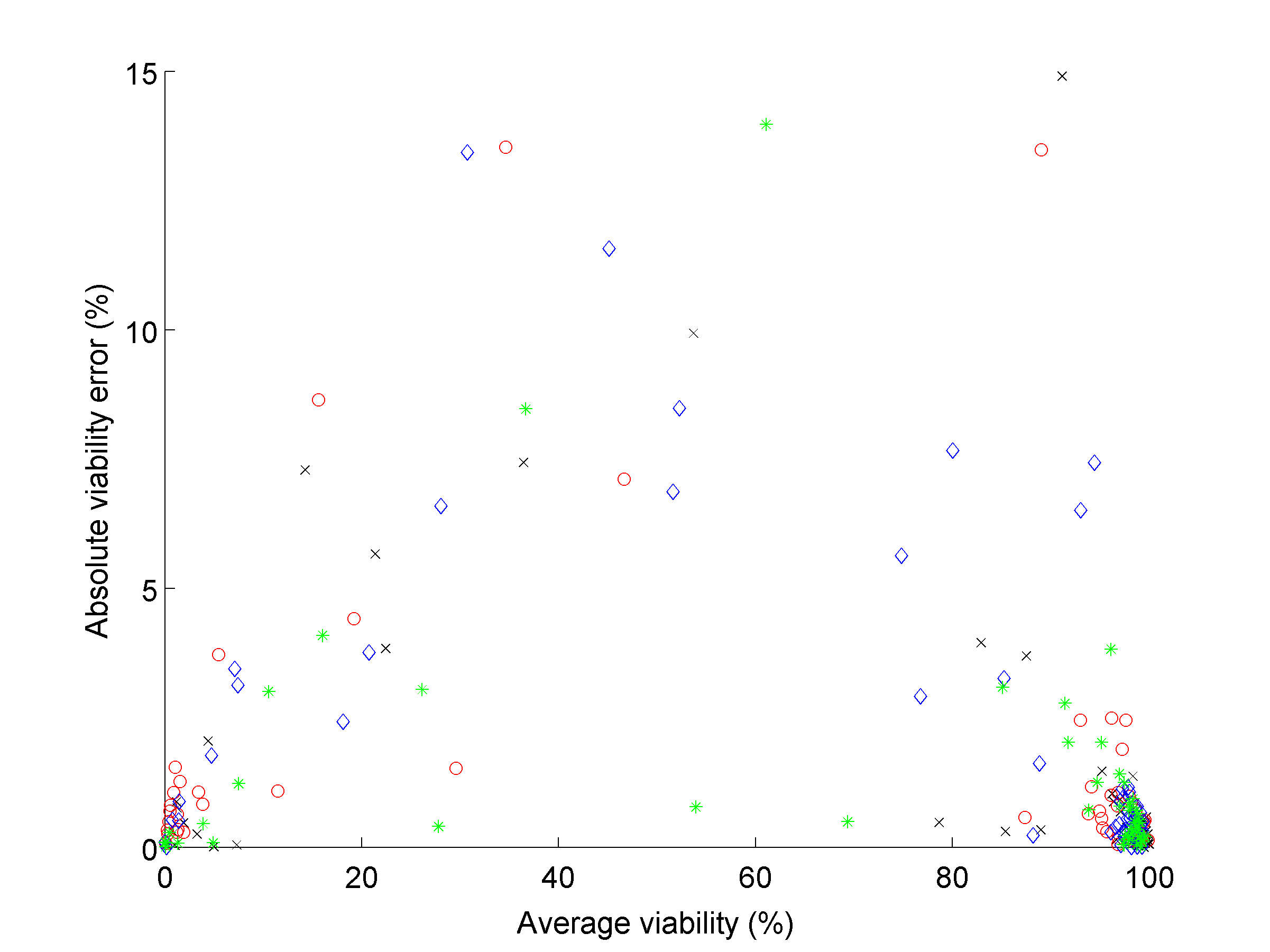


Figure S3-S1: Error on viability (defined here as the difference between replicates) vs. Measured viability (calculated by averaging the replicate values) - Dye/Exposure time: Black crosses: erythrosine/1h; Red circles: erythrosine/6h; Black diamonds: FDA/1h; Green stars: FDA/6h.


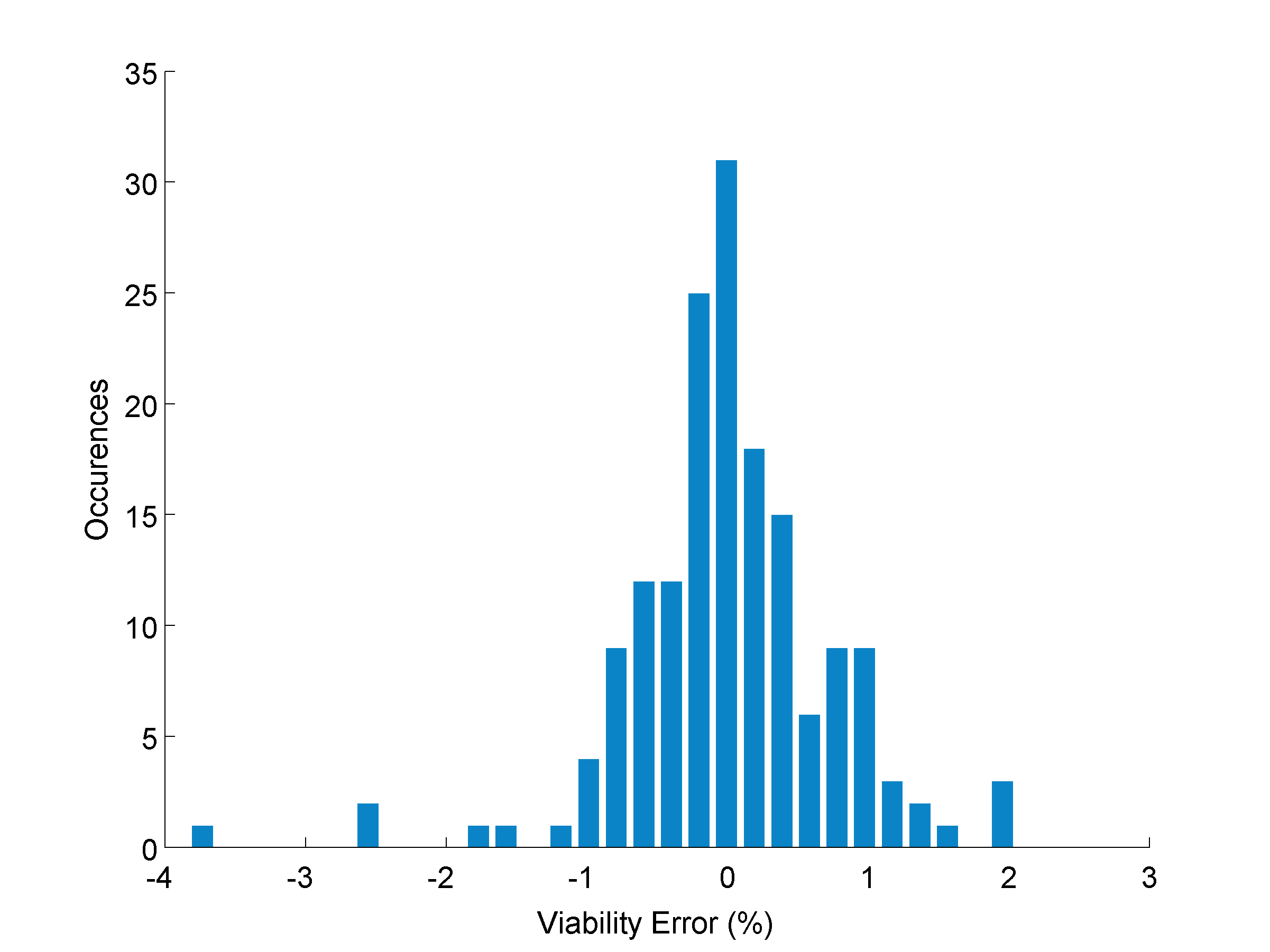


Figure S3-S2: Distribution of error for viability measurements between 0-5% or between 95-100%.


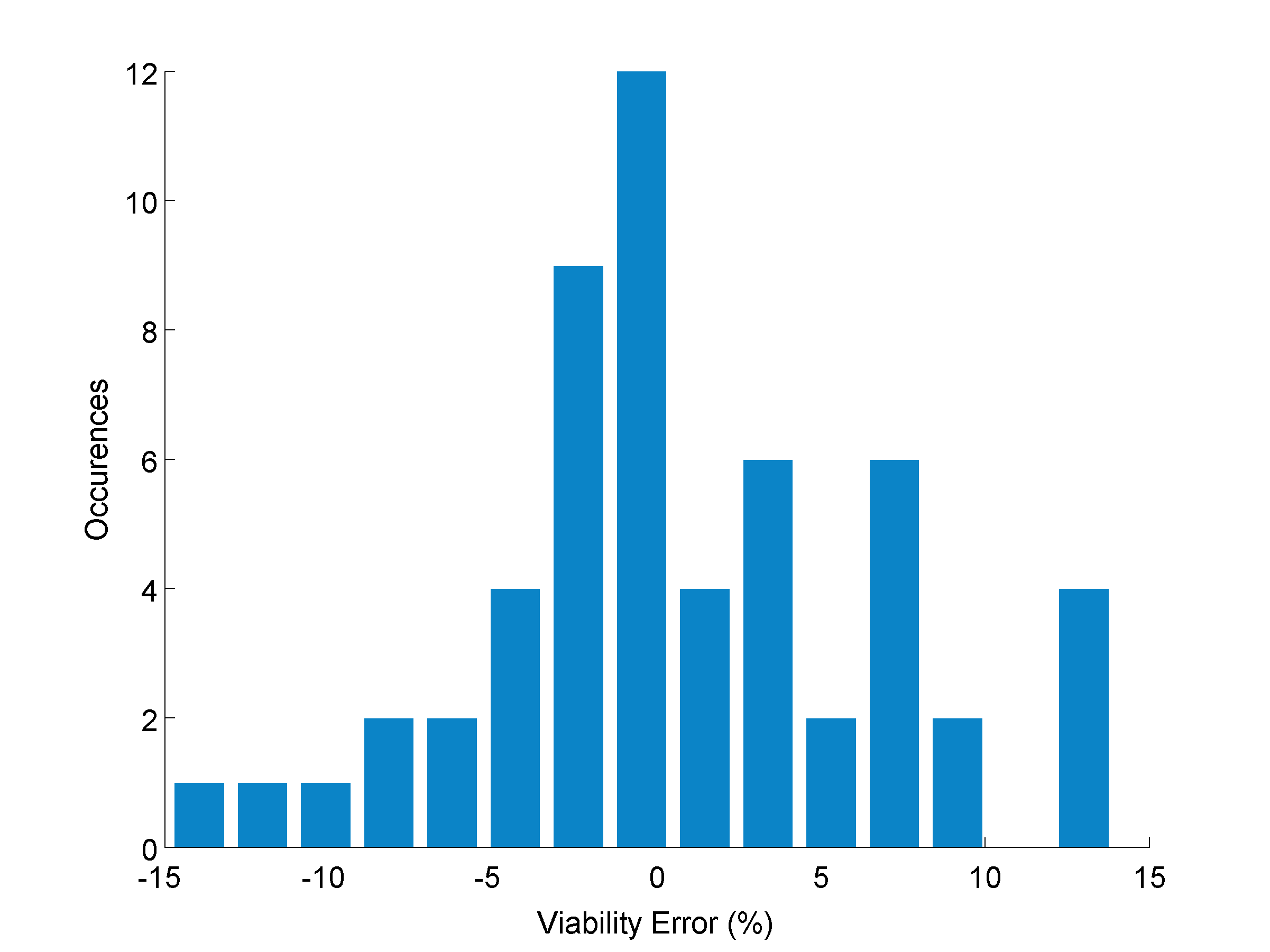


Figure S3-S3: Distribution of error for viability measurements between 5-95%.

Measured viability *V_meas_* can be expressed as the sum of the 'true' value of viability *V_true_* and an error *ε*, which is normally distributed with a standard deviation *σ_ε_*:

$V_{meas}=V_{true}+\varepsilon$

The standard deviation *σ_ε_* can be expressed directly from *σ* as follows:

$$\sigma=\frac{\sigma_{\varepsilon}}{\sqrt{2}}$$

Finally, the confidence interval at 95% *CI_95_* on each viability measurement was expressed as a function of the number of replicates *n* as follows:

- $CI_{95}=\frac{0.90}{\sqrt{n}}$ when viability was either between 0-5% or between 95-100% (*N* = 165)

- $CI_{95}=\frac{7.98}{\sqrt{n}}$ when viability was between 5-95% (*N* = 61)

Based on these confidence intervals, uncertainty on the Weibull parameters *λ* and *n* was determined through Monte-Carlo simulations as follows:

- For each simulation, a 'random' data set of viability vs. time was generated based on the knowledge of the measured viability and associated levels of confidence at 95%;
- The parameters *λ* and *n* were then determined for each of these data sets;
- The standard deviations of the resulting values of *λ* and *n* were then used to determine confidence intervals on these two parameters.

The same Monte-Carlo approach was used to determine confidence intervals on the parameters *α* and *T_0_* defined along the thermal dose concept (see main manuscript for details).

## Photosynthetic activity

A strategy similar to the case of viability was followed to determine confidence intervals on model parameters for photosynthetic activity. Figure S3-S4 shows that the error on the photosynthetic activity (defined here as the difference between duplicate values) was normally distributed.


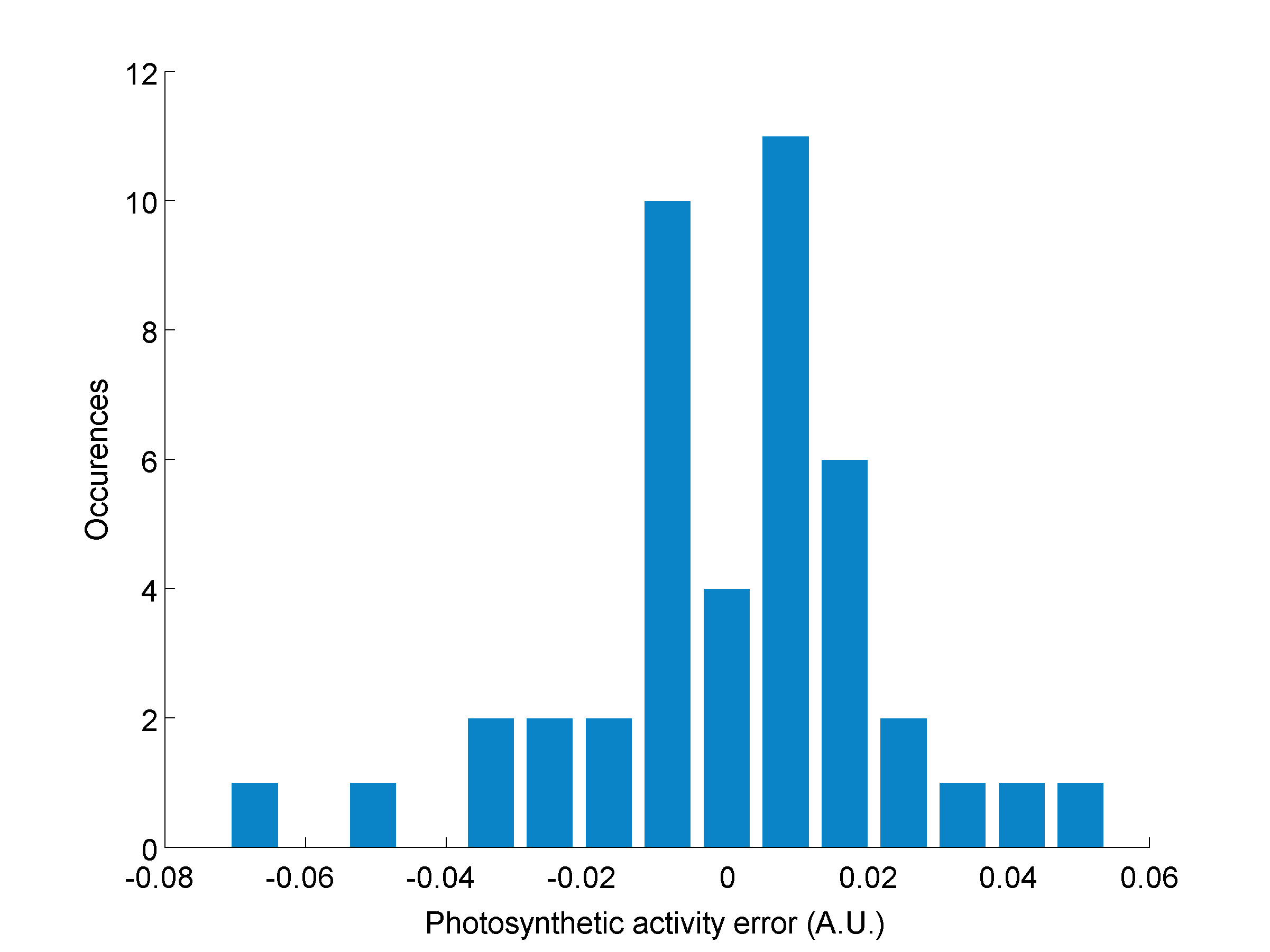


Figure S3-S4: Distribution of error on photosynthetic activity measurements (defined here as the difference between duplicate values)

Based on the same approach than the approach used for viability measurements, the confidence interval on photosynthetic activity measurements was determined as follows (from 44 duplicate measurements):

$CI_{95}=\frac{0.0293}{\sqrt{n}}$

where *n* is the number of replicates. The unit of this confidence interval was arbitrarily set by the PAM analyser and is in the unit of 'electron transfer rate' per unit light intensity (in μmol m^-2^ s^-1^).

Monte Carlo simulations (1000 simulations for each parameter) were then used to determine confidence intervals on model parameters as described in section 3.1.

# Comparison of Weibull and first-order fits to experimental data

Figure S4-S1 shows that the first-order model was unable to satisfying fit the decrease of viability with time of exposure to heat, on the contrary to the Weibull model.


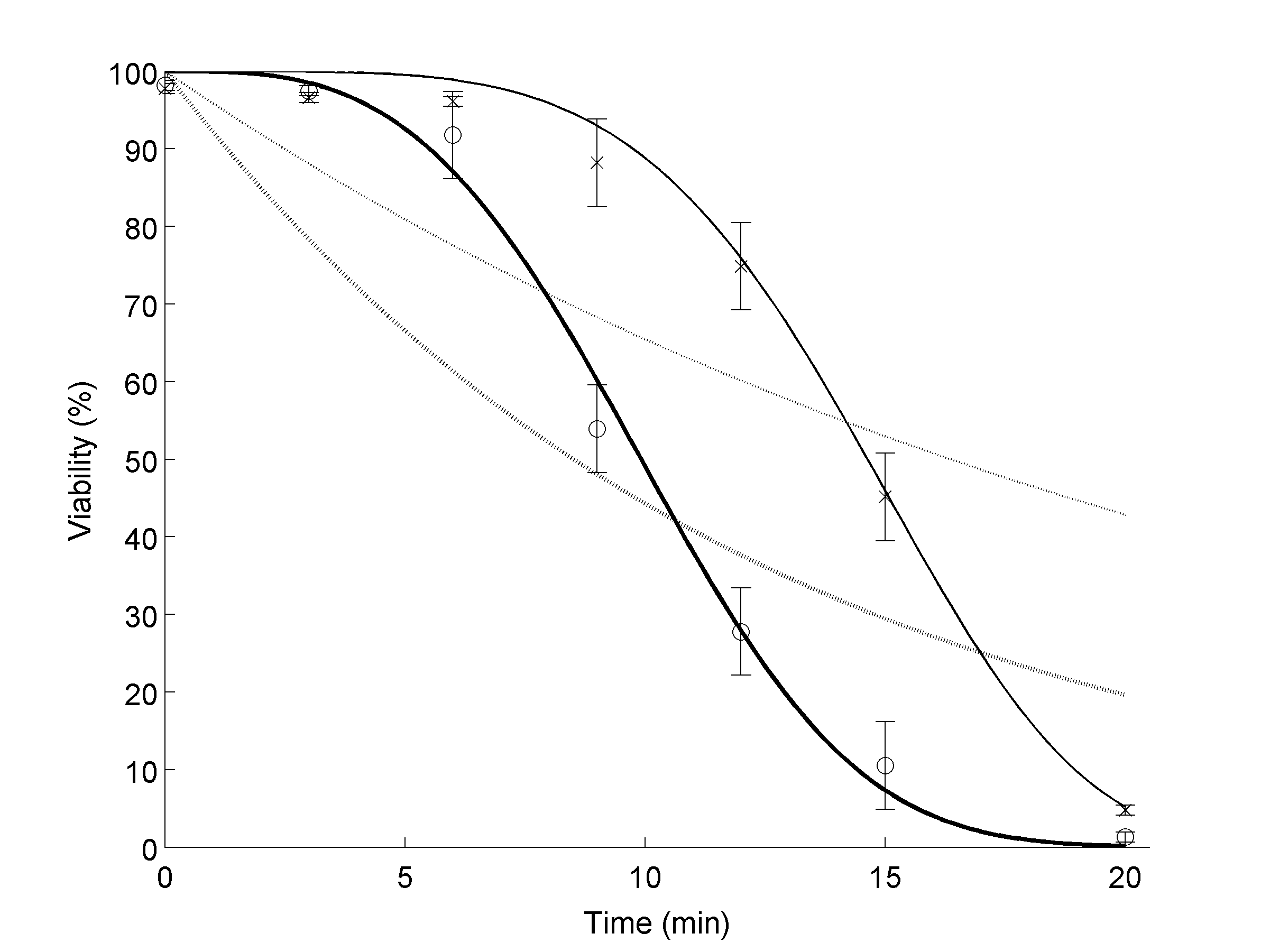


Figure S4-S1: Evolution of algal viability with time of exposure to a temperature of 50^o^C measured with FDA 1 h (crosses) and 6 h (circles) after heat exposure (Dot lines: first-order model; Plain lines: Weibull model). Similar results were obtained at other heating temperatures (data not shown).

# Viability results 6 hours after heat exposure


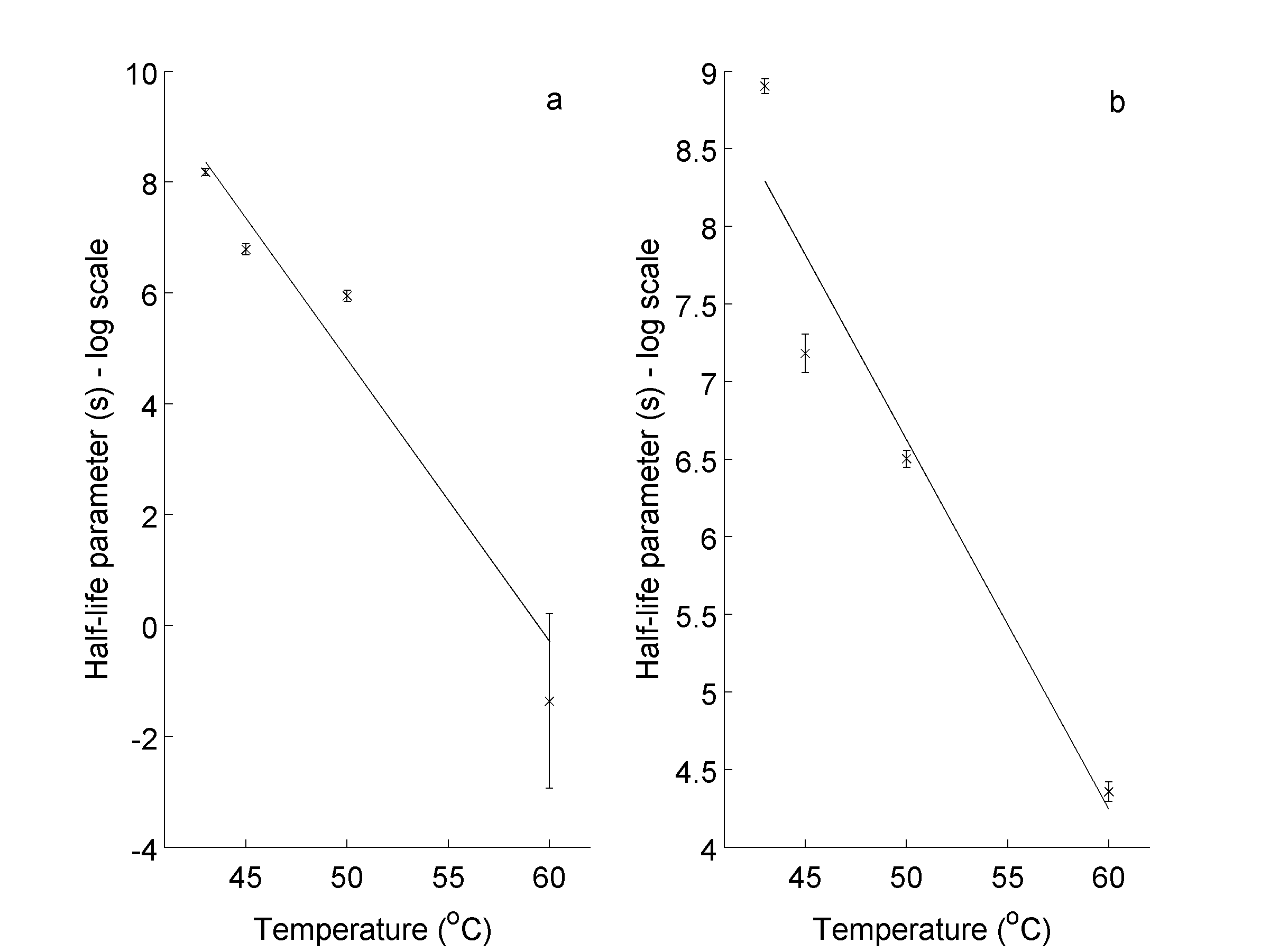


Figure S5-S1: Evolution of Weibull *λ* parameter with temperature when erythrosine (a) and FDA (b) were used to measure viability 6 h after heat exposure.


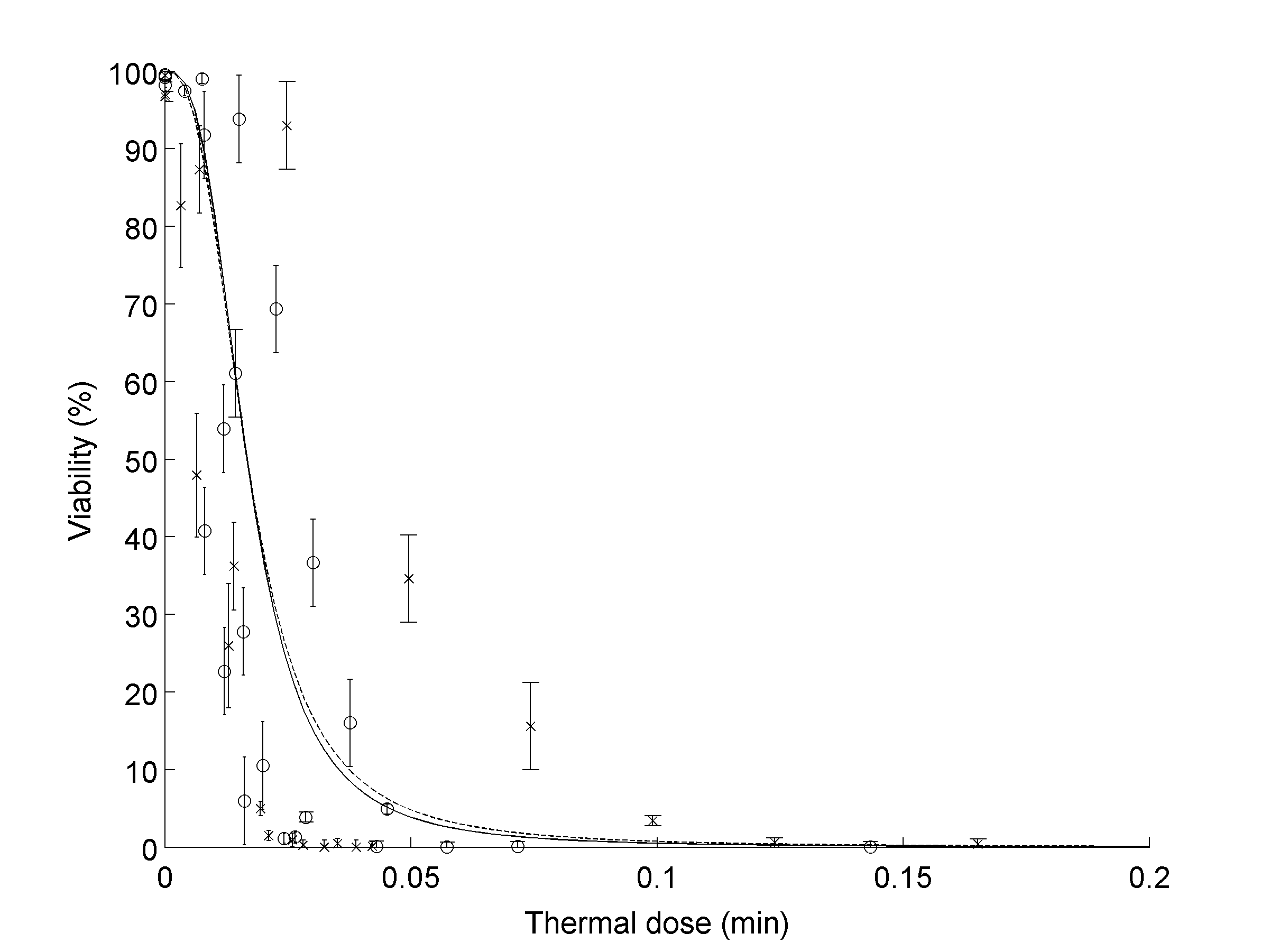


Figure S5-S2: Evolution of the viability with the thermal dose as defined in Equation 9 in the main manuscript with the Weibull model (Crosses/plain line: measurements/prediction with erythrosine; Circles/dash-line: measurements/prediction with FDA) - Viability was measured 6 h after heat exposure - See Table S5-S1 for parameters values.

Table S5-S1: Values of thermal dose model parameters (see main manuscript for model parameters description) when viability was measured 6 h after heat exposure. Values in parenthesis are 95% confidence intervals.

|  | Marker | *a_V_/a_A_* (^o^C^‑1^) | *T_0,V_/T_0,A_* (^o^C) | Shape factor *n* |
| --- | --- | --- | --- | --- |
| Viability | Erythrosine | -0.510 (+/-0.116) | 59.6 (+/-3.3) | 2.85 (+/-1.99) |
|  | FDA | -0.238 (+/-0.005) | 77.8 (+/-0.6) | 2.71 (+/-0.27) |

# Evolution of the photosynthetic activity during kinetic studies

Figure S6-S1 shows the variation of the photosynthetic activity with time when cells were exposed to different temperatures. The experiments at 45^o^C were repeated three times and small variations observed in Figure S6-S1 are most likely due to experimental error (e.g., water bath temperature). The slight decrease observed at a temperature of 41^o^C was most likely due to experimental uncertainties.


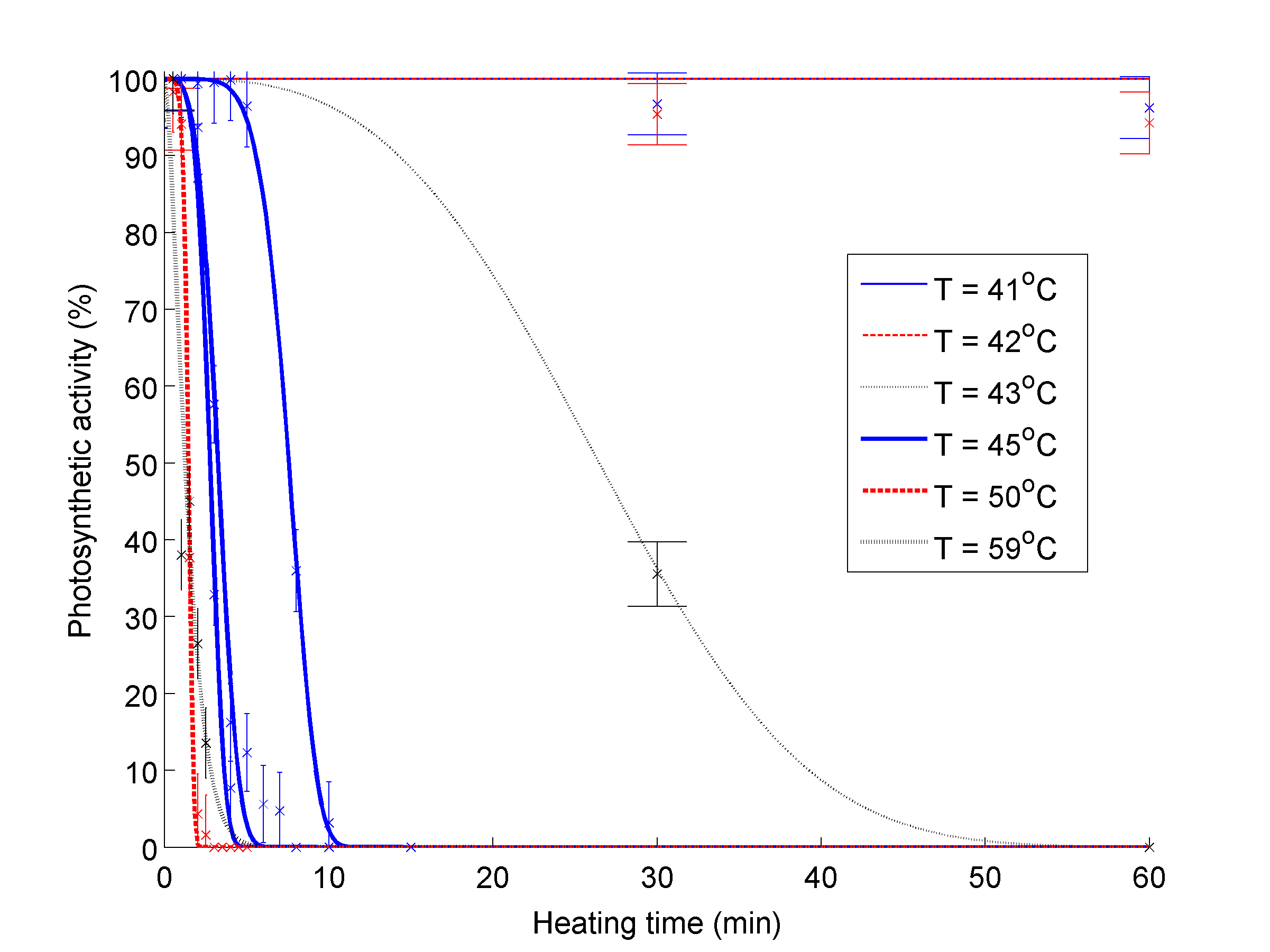


Figure S6-S1: Evolution of measured photosynthetic activity with time of exposure to high temperatures (crosses: experimental data; line: Weibull model) - As the slight drop of the measured photosynthetic activity at 41 and 42^o^C was most likely due to experimental errors, the fitting at these temperatures is a flat line.


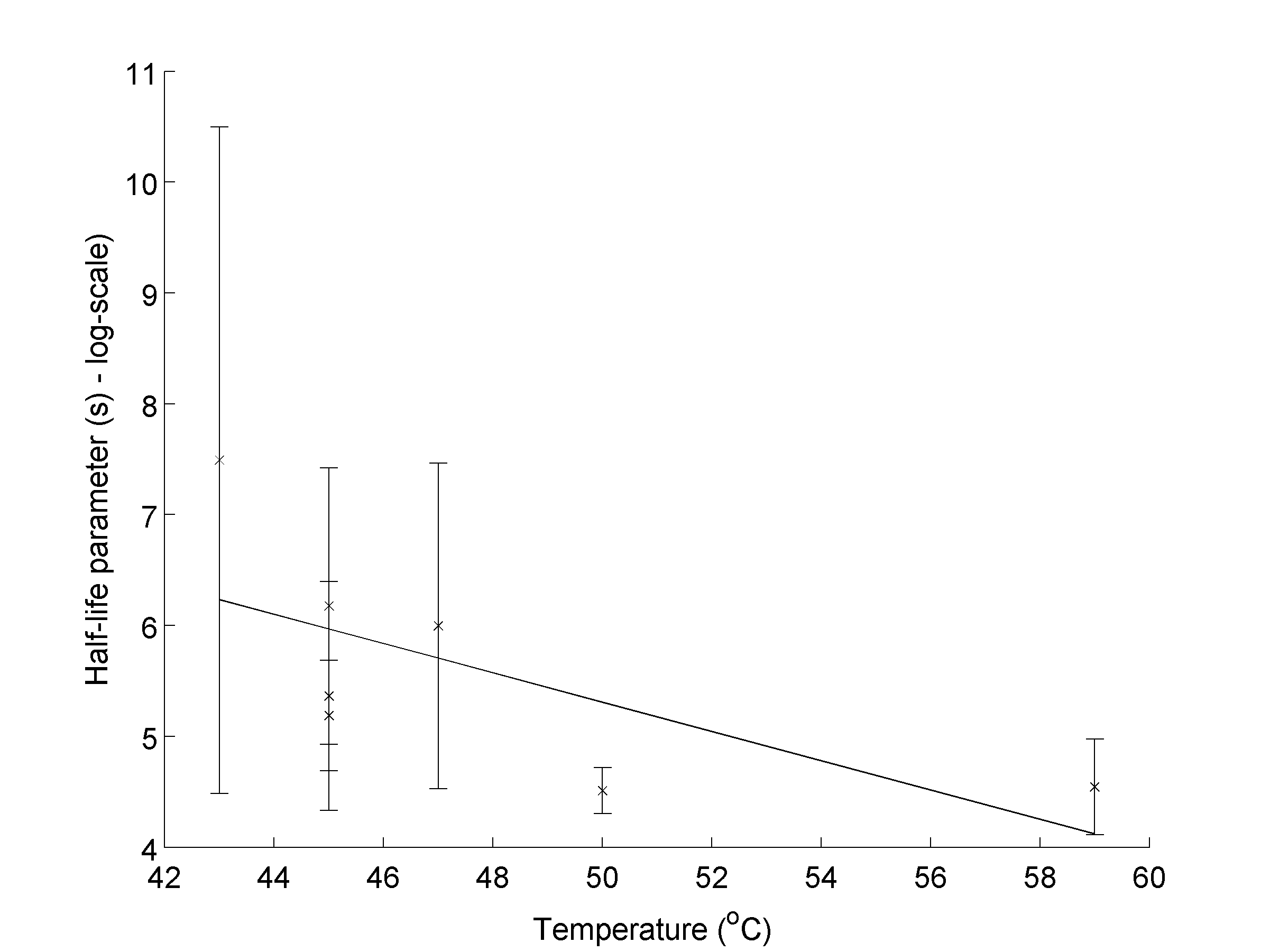


Figure S6-S2: Evolution of the Weibull coefficient *λ* with temperature for photosynthetic activity (Crosses: experimental data; Line: linear regression).
